# Supplementary material for: Enhancement of the electrochemical properties of commercial coconut shell-based activated carbon by H2O dielectric barrier discharge plasma
Source: R Soc Open Sci. 2019 Feb 13;6(2):180872. doi: 10.1098/rsos.180872 (PMC6408405; doi:10.1098/rsos.180872)
Supplement: XPS spectra of CSAC and HCSAC;Electrochemical performance data of samples prepared under different time and power [file rsos180872supp1.docx]

Supporting Information

**Enhancement** **on the electrochemical properties of commercial coconut shell-based activated carbon by H_2_O dielectric barrier discharge plasma**

Xin Wang^1,2^, Xiaoyan Zhou^1,2*^, Weimin Chen^1,2*^, Minzhi Chen^1,2^ and Chaozheng Liu^1,2^

^1^College of Materials and Engineering, Nanjing Forestry University, Nanjing, 210037, China.

^2^Jiangsu Engineering Research Center of Fast-growing Trees and Agri-fiber Materials, Nanjing 210037, China

*Corresponding author:

E-mail address: [zhouxiaoyan@njfu.edu.cn](mailto:zhouxiaoyan@njfu.edu.cn) (XY. Zhou); [cwmwood@163.com](mailto:cwmwood@163.com) (WM. Chen).





Fig. S1 XPS spectra of CSAC and HCSAC


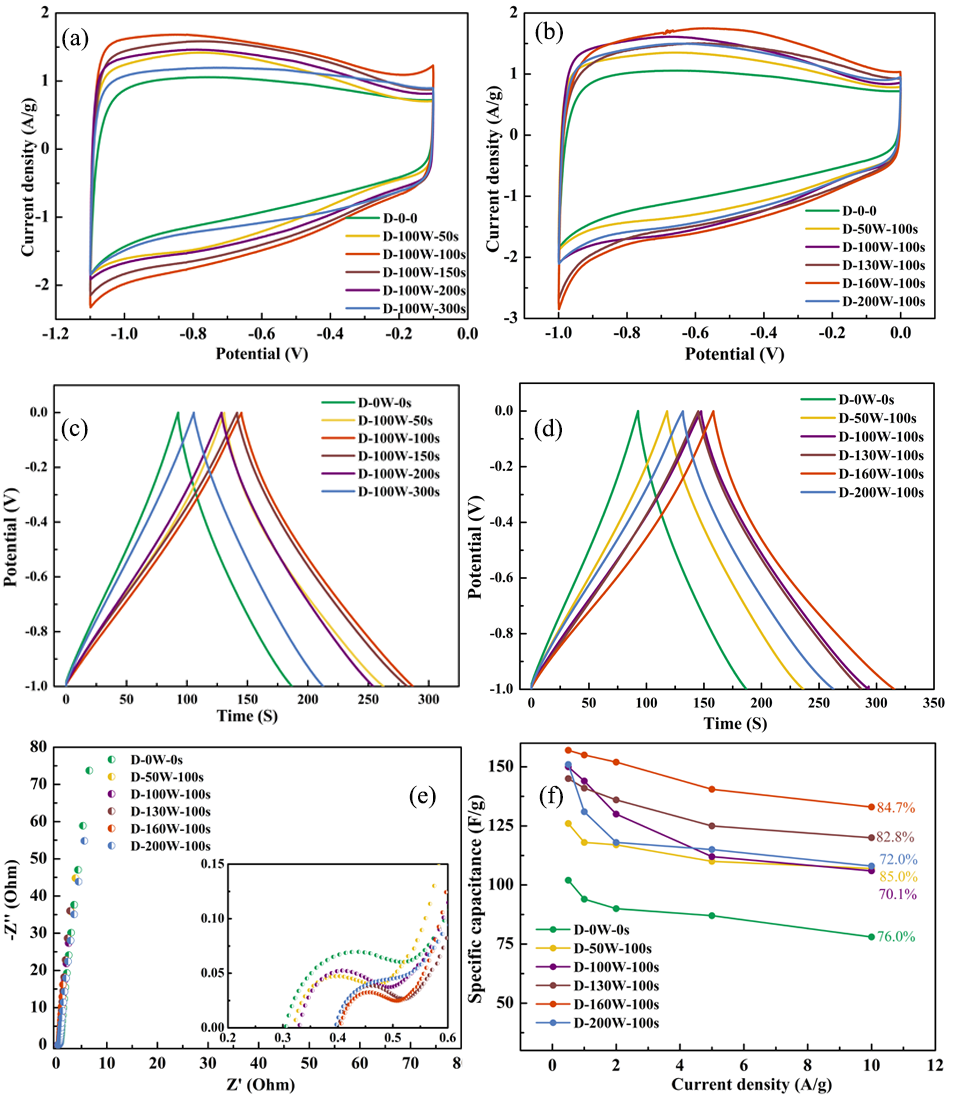


Fig. S2 (a-b) CV curves of CSAC and CSAC modified with DBD H_2_O plasma in a three-system with 6 M KOH aqueous electrolyte at the scan rate of 10 mV s^-1^. (c-d) GCD curves of CSAC and CSAC modified with DBD H_2_O plasma in a three-electrode system with 6 M KOH aqueous electrolyte at the current density of 1 A g^-1^. (e) Nyquist plots of CSAC and CSAC modified at different power electrodes. The inset is the detail with enlarged scale (f) Rate capability of CSAC and CSAC modified at different power at the current density from 0.5 to 10 A g^-1^.
